# Optimization of Extended-Release ZL-004 Nanosuspensions for In Vivo Pharmacokinetic Study to Enhance Low Solubility and Compliance

**DOI:** 10.3390/molecules24010007

**Published:** 2018-12-20

**Authors:** Chengyue Guo, Yanna Chen, Junzhe Zhu, Jiaxin Wang, Ying Xu, Hansen Luan, Hao Wang

**Affiliations:** National Pharmaceutical Engineering Research Center, China State Institute of Pharmaceutical Industry, Shanghai 201203, China; chengyueguo@outlook.com (C.G.); chyann72@hotmail.com (Y.C.); zjz.miffy@foxmail.com (J.Z.); wangjiaxin0108@163.com (J.W.); xuying0613wh@163.com (Y.X.); luanhansen@sina.com (H.L.)

**Keywords:** nanosuspensions, extended-release, particle size, free stabilizers, dissolved drug

## Abstract

ZL-004, a promising small molecule that increases white blood cell counts, was developed for extended-release nanosuspensions to improve low solubility and compliance of patients. In vivo pharmacokinetic studies of nanosuspensions with different particle sizes and administration volumes were conducted. Unexpectedly, C_max_ of NS-PC-L (1156 nm) was 1.3 fold higher than NS-PB-L (836 nm), and area under plasma concentration-time curve (AUC) was similar. It suggested that in vivo behavior of nanosuspensions was influenced significantly by the original dissolved drug, which did not only rely on the particle size but also the amount of the free stabilizers. In addition, smaller administration volume (0.1 mL) achieved significantly lower C_max_ and AUC than the higher volume (0.5 mL), due to the reduced amount of dissolved drug. DSC and XPRD demonstrated that the crystal forms of nanosuspensions prepared by the precipitation method and high-pressure homogenization were similar; therefore, in vivo behaviors did not show significant differences. An additional 0.15% PEG 4000 enhanced the redispersity and maintained the particle size for 3 months. Finally, a nanosuspensions with the desired initial release was achieved, which lasted approximately 32 days steadily after a single dose. AUC and t_1/2_ were 161.2 fold and 22.9 fold higher than oral administration.

## 1. Introduction

**Figure 1:**
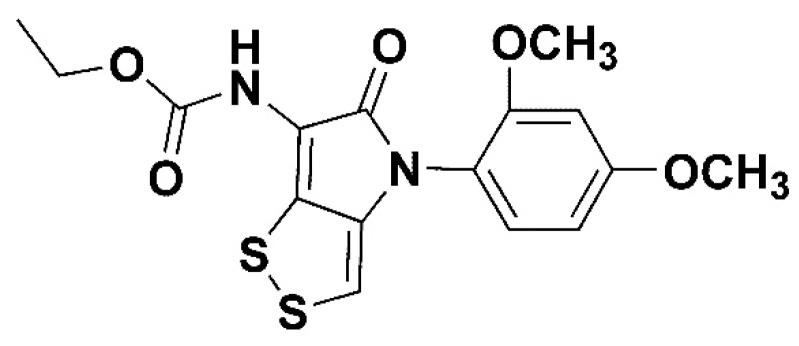
Chemical structure of ZL-004.

ZL-004 (Figure 1) is the derivative of dithiolopyrrolones, which are a class of natural products possessing 4H-[1,2]dithiolo[4,3-b]pyrrol-5-one skeleton [1]. The dithiolopyrrolones have been mostly reported to have antibacterial activity [2,3,4]. Surprisingly, ZL-004 was found to exhibit antileukopenia activity, which could increase white blood cell counts in normal mice and chemotherapy-induced mice significantly [1,5]. Currently, the main drug to increase white blood cell counts for chemotherapy-induced patients is protein: recombinant human granulocyte colony stimulating factor (rhG-CSF) [6,7,8]. Therefore, ZL-004 as a small molecular compound is worth further investigation and development. However, further investigations are impeded by the low solubility of ZL-004 (<1 μg/mL). It is essential to improve the low solubility and bioavailability of ZL-004. Meanwhile, in order to improve the compliance of cancer patients and avoid daily injections, in this study we aim to develop ZL-004 for extended-release nanosuspensions.

Extended-release nanosuspensions for intramuscular use are a kind of drug form that drug crystals disperse in a liquid medium (typically water), and the size of crystals is in nanoscale [9]. Nano-sized particles could improve bioavailability by increasing dissolution rate and solubility according to the Noyes–Whitney equation and the Ostwald-Freundlich equation [10]. Meanwhile, as the essential components of nanosuspensions are only nano-sized drug crystals and stabilizers like surfactants, the drug concentration is high [11]. Extended-release nanosuspensions work by acting as a reservoir, which slowly delivers the dissolved drug into the systemic circulation; hence, it could improve compliance of patients [12]. Unlike liposome, microsphere, and micelle, nanosuspensions have no carriers, the in vivo release behavior of nanosuspensions depends on the properties of the drug, crystal, stabilizers, and medium [13]. Therefore, particle size plays an important role; smaller particles usually achieve higher bioavailability. [14]. However, it was reported that intramuscularly long-acting paliperidone palmitate nanosuspension A (1041 nm) achieved higher bioavailability than the nanosuspension B (505 nm), AUC_0−t_ and C_max_ of nanosuspension A was 2.0-fold and 1.8-fold higher than nanosuspension B, the reason was not elaborated [15]. Therefore, to investigate the effect of particle size on in vivo release and choose a proper particle size, a pharmacokinetic study of ZL-004 nanosuspensions with different particle sizes (about 400 nm, 800 nm, and 1200 nm) was conducted. Meanwhile, another easily neglected influencing factor, the effect of administration volume on in vivo release was evaluated. 

The methods for preparing nanosuspensions are classified as top-down and bottom-up [16]. The top-down method is reducing large particles into small ones by met-milling or high-pressure homogenization; the bottom-up method is crystallizing by methods such as precipitation, spray drying, and freeze drying [17,18]. The top-down method is used widely for large-scale industrial production [19,20]. As the principles of forming nanosuspensions are different, nanosuspensions are prepared by both of the methods in this study, and pharmacokinetic studies are conducted respectively to make a comparison. Moreover, as the particle size of nanosuspensions is important for the in vivo behavior [21], the stability of the particle size and redispersity of the nanosuspensions in a liquid state for at least 3 months storage were investigated.

## 2. Results and Discussion

### 2.1. Preparation

#### 2.1.1. Precipitation Method

**Figure 2:**
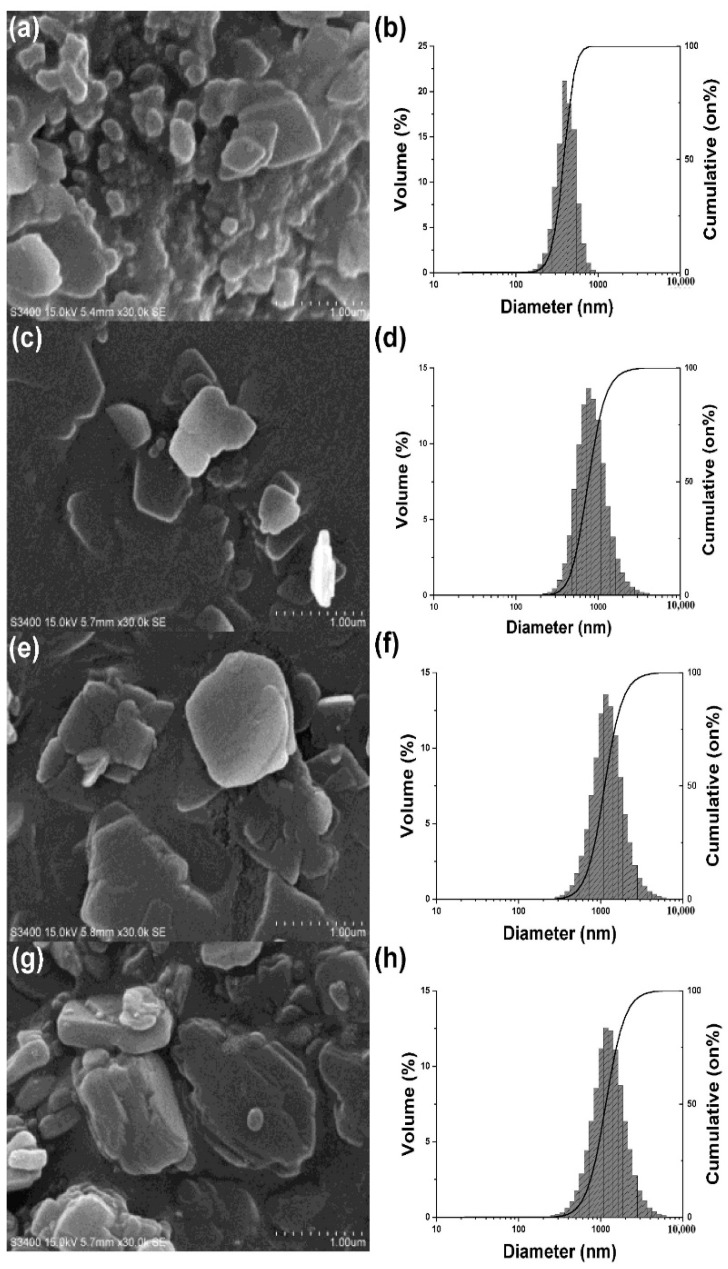
SEM images of NS-PA (**a**), NS-PB (**c**), NS-PC (**e**) and NS-Hα (**g**). Particle size of NS-PA (**b**), NS-PB (**d**), NS-PC (**f**) and NS-Hα (**h**) measured by laser diffraction instrument.

The particle size (D_50_) of NS-PA (4 min-injection, 4 °C), NS-PB (2 min-injection, 4 °C), and NS-PC (2 min-injection, 10 °C) was 424 nm, 812 nm, and 1185 nm respectively. The specific data of particle size is shown in Table 1 and Figure 2. The particle size of NS-PA reduced as the time of injection and ultrasonic increased due to the increased erosion and diffusion compared with NS-PB. In addition, the particle size of NS-PC increased compared with NS-PB due to the decline of the supersaturation, which was caused by the increment of solubility as the temperature increased. After freeze drying, the particle sizes of NS-PA-L, NS-PB-L, and NS-PC-L were 397 nm, 836 nm, and 1156 nm, the particle sizes of NS-PB-S and NS-PC-S were 806 nm and 1225 nm, which were similar to the particle sizes before freeze drying. The specific particle sizes are shown in Table 2. The structure of the nanosuspensions after freeze drying was cubic without collapse and shrink, which could be redispersed rapidly.

#### 2.1.2. High-Pressure Homogenization Method

The particle size of NS-Hα (25,000 psi, 27 min, 0.5% Tween-80), NS-Hβ (25,000 psi, 12 min, 1% Tween-80), and NS-Hγ (15,000 psi, 27 min, 0.5% Tween-80) was 1134 nm, 1120 nm, and 1467 nm respectively. The particle size reduced more rapidly by higher pressure with a higher concentration of Tween-80, which is shown in Figure S1. NS-Hβ with more surfactants absorbed in the particles helped prevent the aggregation and enhance the fluidity, which led to an increase in efficiency compared with NS-Hα. The particle sizes of NS-Hα-1 and NS-Hα-2were 1211 nm and 1144 nm, which were similar to the particle sizes before post treatment. The specific particle sizes are shown in Table 2.

In general, the required liquid volume of the homogenizer was large; at least 40 mL was needed. However, the lowest volume of the precipitation method was not limited. Furthermore, the yield of the homogenizer was low due to the pipelines. However, the equipment of the precipitation method (a stirrer combined ultrasonic probe) was simple, and the loss was less. Therefore, the precipitation method was more suitable for precious API (active pharmaceutical ingredients) in the lab despite requiring freeze drying. As we used little API in this study, nanosuspensions were first prepared and optimized by the precipitation method, then the comparison of two methods was conducted to facilitate the design and preparation of the formulations prepared by the high-pressure homogenization method for large-scale production.

### 2.2. In Vitro Release Study

**Figure 3:**
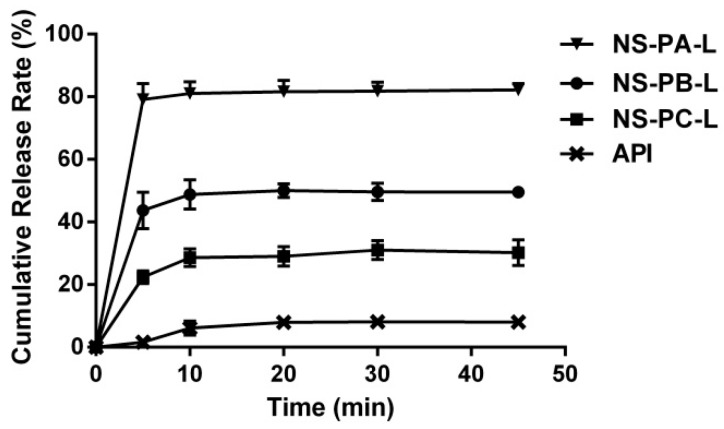
In vitro cumulative release curves of NS-PA-L, NS-PB-L, NS-PC-L, and API (Mean ± SD, *n* = 3).

In vitro cumulative release curves of NS-PA-L, NS-PB-L, NS-PC-L, and API are shown in Figure 3. All of the curves had reached the platform at 45 min. The cumulative release of NS-PA-L, NS-PB-L, NS-PC-L, and API were 82.17%, 49.55%, 30.20%, and 8.02% respectively. Owing to the higher surface area, smaller particles showed significantly enhanced dissolution rate and solubility [22]. 

### 2.3. Oral Administration 

**Figure 4:**
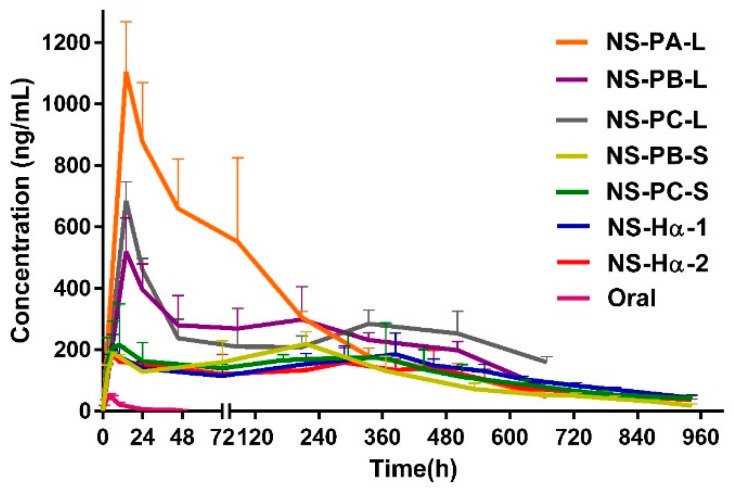
Average plasma concentration-time curve.

It was reported that leukocyte-increasing activity was achieved when ZL-004 was administrated intragastrically at the dose of 10 mg/kg by suspending in water with 0.5% CMC-Na and 4% Tween-80 [23]. In this study, the same dose of ZL-004 was administrated in the same way, the plasma concentration-time profile is shown in Figure 4, and the pharmacokinetic parameters are shown in Table 3. AUC was 0.6 µg·h/mL, t_1/2_ was 11.0 h, C_max_ was 48.6 ng/mL. As the accurate therapeutically effective concentration was indeterminate, in order to achieve leukocyte-increasing activity, the target plasma concentration for nanosuspensions in this study was not lower than 50 ng/mL. 

### 2.4. In Vivo Pharmacokinetic Study with Different Particle Sizes

The plasma concentration–time profiles of NS-PA-L, NS-PB-L, and NS-PC-L with increasing particle size are shown in Figure 4, the pharmacokinetic parameters are shown in Table 3. All of T_max_ was 14.5 h. t_1/2_ of NS-PA-L, NS-PB-L, and NS-PC-L were 170.5 h, 222.0 h, and 406.8 h respectively, which increased significantly as the particle size increased. C_max_ of NS-PA-L and NS-PB-L were 1104.7 ng/mL and 518.9 ng/mL; AUC of NS-PA-L and NS-PB-L were 192.3 µg·h/mL 153.2 µg·h/mL. It indicated that C_max_ and AUC reduced as the particle size increased from 397 nm to 836 nm. However, C_max_ of NS-PC-L (1156 nm) was 685.5 ng/mL, which was 1.3 fold higher than NS-PB-L. AUC was 163.0 µg·h/mL, which was similar to NS-PB-L. 

**Figure 5:**
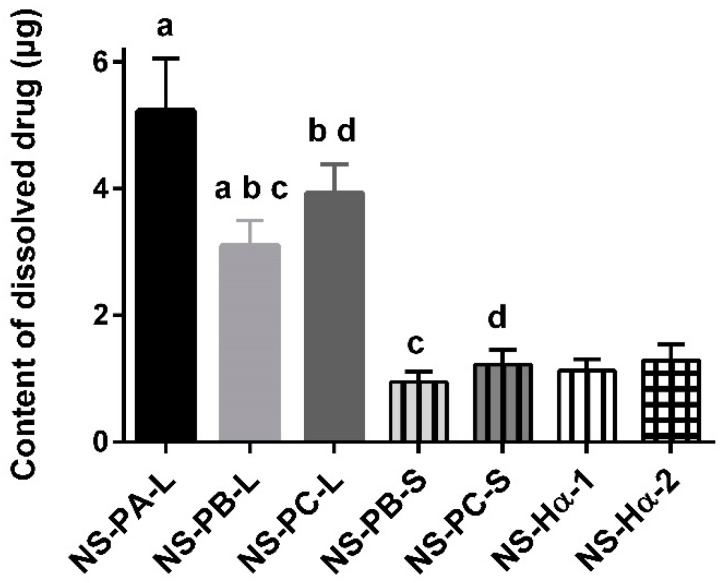
The content of dissolved drug (Mean ± SD, *n* = 3). ^a,b,c,d^ *p* < 0.05.

C_max_ and AUC of NS-PB-L were significantly lower than NS-PA-L due to the larger particle size. However, the marginally higher C_max_ of NS-PC-L was significant compared to NS-PB-L, and AUC did not show a significant difference, despite NS-PC-L achieving lower dissolution rate and solubility due to the larger particle size. It indicated that the in vitro–in vivo correlation was poor [24]. C_max_ and AUC were not only influenced by the particle size. According to Figure 4, all the formulations peaked at the same hour and then declined sharply; after 72 h the curves began to decline more gently. Furthermore, the change of C_max_ was consistent with the change of the content of dissolved drug. The dissolved drug of NS-PB-L was 1.7 fold lower than NS-PA-L and 1.2 fold lower than NS-PC-L (Figure 5). It suggested that the rapid initial release (0 to 72 h) was influenced by the content of the dissolved drug significantly, which permeated membranes into blood rapidly without the dissolution process. It was easily neglected that the content of dissolved drug was not only affected by the particle size but also the amount of free stabilizers [25]. The stabilizers consisted of the ones absorbed to the surface area of crystals and the free ones. When the surface area reduced as the particle size increased, the free stabilizers increased at the same time, which increased the content of dissolved drug by forming micelles. Ultimately, it transpired that the content of dissolved drug of NS-PC-L was higher than NS-PB-L and NS-PC-L achieved an enhanced initial release. Then, after the original dissolved drug rapidly entered into the blood, the release was limited to the particle size. The increasing particle size reduced the dissolution rate, which led to significantly increased t_1/2_. t_1/2_ of NS-PC-L was 1.7 fold higher than NS-PB-L and t_1/2_ of NS-PB-L was 1.4 fold higher than NS-PA-L. Only NS-PA-L expressed a plasma concentration less than the target (50 ng/mL) at 27 days after administration. Therefore, this research was terminated prematurely, and the particle size of about 400 nm was abandoned. Furthermore, NS-PB-L and NS-PC-L needed optimizations for reducing the overly high initial release. 

Generally, the optimization of particle size is dependent on the therapeutic purpose. Particle size is usually reduced to improve bioavailability due to the increased solubility and dissolution rate. To achieve an extended and steady release, a larger particle size is ideal; to achieve a fast release and higher plasma concentration, a smaller particle size is required. However, the in vivo behavior is also dependent on the amount of free stabilizers, especially for the initial release. A larger particle size could achieve similar or even higher initial release and bioavailability compared with a smaller particle size when the total stabilizer concentration is the same. The effect of the free stabilizers must be seriously taken account of when selecting the particle size.

### 2.5. In Vivo Pharmacokinetic Study with Different Administration Volume

The plasma concentration-time profiles and pharmacokinetic parameters of NS-PB-S and NS-PC-S are shown in Figure 4 and Table 3. Compared with NS-PB-L and NS-PC-L, the dose volume of NS-PB-S and NS-PC-S reduced from 0.5 mL to 0.1 mL, and the concentration increased 5 fold to maintain the dose level. A smaller administration volume of NS-PB-S and NS-PC-S led to a significant decrease of the initial release. C_max_ (0 to 72 h) of NS-PB-S and NS-PC-S were 189.4 ng/mL and 232.9 ng/mL, which was 2.7 fold and 2.9 fold lower than NS-PB-L and NS-PC-L. AUC of NS-PB-S and NS-PC-S were 98.5 µg·h/mL and 110.2 µg·h/mL, which was 1.6 fold and 1.5 fold lower than NS-PB-L and NS-PC-L. In addition, the comparison of NS-PB-L and NS-PC-L showed that t_1/2_ of NS-PC-S (266.9 h) was 1.3 fold higher than NS-PB-L (208.8 h); however, AUC and C_max_ were not significantly influenced by the different particle sizes. For intramuscular extended-release nanosuspensions, loading doses and oral supplementation are usually in need of attaining therapeutically effective concentrations rapidly [26,27]. Although NS-PB-L and NS-PC-L achieved rapid initial release and high plasma concentration, overly high drug concentration should be reduced to avoid undesirable side effects when the therapeutic window of ZL-004 is indeterminate. Therefore, the content of dissolved drug of NS-PB-S and NS-PC-S were reduced 3.3 fold and 3.1 fold by reducing the administration volume to decrease C_max_ and AUC. Except for the volume itself, the content of the dissolved drug was also influenced by the amount of free stabilizers. As the Tween-80 concentration of NS-PB-S and NS-PC-S was same as NS-PB-L and NS-PC-L, the concentrated crystals increased the total surface area. Therefore, the free Tween-80 declined, which also reduced the content of the dissolved drug. In addition, C_max_ and AUC of NS-PC-S did not show significant differences from NS-PB-S when the particle size increased from 806 nm to 1225 nm due to the similar content of dissolved drug. The t_1/2_ of NS-PC-S with the larger particle size demonstrated a marginally significant increase compared with NS-PB-S, and the mean days of attaining therapeutically effective plasma concentration increased from 27 days to 32 days. Owing to enhanced therapeutically effective days and facilitation of preparation, NS-PC-S was the proper formulation.

### 2.6. Comparisons of Nanosuspensions Prepared by the Precipitation Method and the High-Pressure Homogenization Method

The SEM images of NS-PA, NS-PB, NS-PC, and NS-Hα are shown in Figure 2. NS-PA, NS-PB, and NS-PC were flakelike and NS-Hα was slightly thicker. The diameters of all the nanosuspensions in the SEM images were consistent with the results measured by the laser diffraction instruments. DSC curves (Figure S2a) showed that the crystal forms of NS-PA, NS-PB, NS-PC, and NS-Hα were the same as the API. XPRD curves (Figure S2b) confirmed that the crystal forms were the same. 

The comparison of in vivo pharmacokinetic profiles of the nanosuspensions with same particle sizes prepared by two methods is shown in Figure 4, the pharmacokinetic parameters are shown in Table 3. C_max_, AUC, and t_1/2_ of NS-Hα-1 were 218.5 ng/mL, 113.1 µg·h/mL, and 241.5 h, which were not significantly influenced by the slightly different thickness of the crystals compared with NS-PC-S. It suggested that the in vivo behaviors of ZL-004 nanosuspensions with the same particle sizes prepared by the precipitation method and the high-pressure homogenization method were similar; NS-Hα-1 was preferred for large-scale production.

### 2.7. Physical Stability

Particle size played a critical part in the in vivo behavior of nanosuspensions; therefore, the stability of the particle size was important. As NS-PA-L, NS-PB-L, NS-PC-L, NS-PB-S, and NS-PC-S were freeze-dried powder, which was redispersed before use, the investigation of the stability of particle size for long-term in the liquid was unnecessary. However, NS-Hα-1 and NS-Hα-2 prepared by the high-pressure homogenization method did not use an organic reagent, which maintained a liquid state without freeze drying. Hence, the liquid stability should be investigated. After storage for 1 month, the particle size of NS-Hα-1 and NS-Hα-2 were stable (Table S1), and all of them could be redispersed. However, after storage for 3 months, NS-Hα-1 (0.5% Tween-80) could not be redispersed; D_50_ increased from 1150 nm to 1506 nm; D_10_ and D_90_ also showed a significant increase. In order to investigate whether higher Tween-80 concentration could enhance redispersity, NS-Hβ (1% Tween-80) with the same particle size was set as a comparison with NS-Hα-1, which could not be redispersed after 3 months either. However, NS- Hα-2 (0.5% Tween-80, 0.15% PEG 4000) was still easily redispersed and the particle size remained stable. The zeta potential of NS-Hα-1 and NS-Hα-2 were −11.9 mV and −16.5 mV respectively. The content of ZL-004 maintained a range of 95.0%–105.0%.

Nanosuspensions with a large surface area, which preferred to aggregate to reduce the great surface energy, were not stable thermodynamically. Meanwhile, according to the Ostwald–Freundlich equation, small particles with a high solubility tended to dissolve and crystallize onto the large particles [28,29,30]. On the other hand, in dynamics, nanosuspensions settled down because of gravity. If the sediment was densely packed, it was difficult to redisperse [31]. Therefore, stabilizers were used to cover the surface of crystals for providing enough electrostatic and steric repulsion to hinder the aggregation and growth [32]. In this study, after 1 month, NS-Hα-1 (0.5% Tween-80) could be redispersed, and the particle size could remain stable. However, after 3 months the sediment, which was a densely caked, could not be redispersed. Meanwhile, the particle size increased significantly. In comparison, after 3 months, NS-Hβ (1% Tween-80) could not be redispersed either. It suggested that the increment of Tween-80 was insufficient to enhance the redispersity; another stabilizer was needed. It is possible that long chains of polymers would link particles together as a bridge. It could lead the nanosuspension to a relatively loose structure, which was easy to be redispersed by agitation [33,34]. Consequently, with an additional 0.15% PEG 4000, NS-Hα-2 could be redispersed easily during the 3 months with stable particle size. The increased zeta potential of NS-Hα-2 also showed that PEG 4000 helped enhance the stability compared with NS-Hα-1 [35]. 

The plasma concentration–time profile and pharmacokinetic parameters of NS-Hα-2 are shown in Figure 4 and Table 3. The content of dissolved ZL-004 of NS-Hα-2 was similar to NS-Hα-1. Therefore, C_max_, AUC, and t_1/2_ were 209.7 ng/mL, 96.9 µg·h/mL, and 252.3 h, which were similar to NS-Hα-1. It suggested that the additional 0.15% PEG 4000 did not affect in vivo behavior significantly. Finally, NS-Hα-2 achieved steady and therapeutically effective concentration values ranging from 51.2 ng/mL to 186.0 ng/mL, which lasted approximately 32 days after a single dose. AUC and t_1/2_ were 161.2 fold and 22.9 fold higher than oral administration; nanosuspensions showed significantly enhanced bioavailability and compliance.

## 3. Materials and Methods

### 3.1. Materials

ZL-004 was synthesized by Guoping Wang team from National Pharmaceutical Engineering and Research Center (Shanghai, China). Tween-80 was purchased from J&K Scientific.Ltd (Beijing, China). PEG 4000 and CMC-Na were purchased from Shanghai Chineway Pharmaceutical Tech. Co. Ltd. (Shanghai, China). Tetrahydrofuran (THF) was purchased from Sinopharm Chemical Reagent Co. Ltd. (Shanghai, China). 

Male Sprague-Dawley (SD) rats (280–300 g) were purchased from the Shanghai Super-B&K Laboratory Animal Corporation Ltd. (Shanghai, China). All the studies were performed in accordance with the Ethical Guidelines for Investigations in Laboratory Animals and were approved by the National Pharmaceutical Engineering and Research Center (2017-0325-001).

### 3.2. Method

#### 3.2.1. Preparation

##### Precipitation Method

Tween-80 (0.5%, *m*/*v*) was dissolved into 20 mL bidistilled water. ZL-004 (120 mg) was dissolved into 4 mL THF. The water solution was cooled to 4 °C for preparing NS-PA and NS-PB, and 10 °C for preparing NS-PC. The THF solution was injected into the water a syringe, and stirring, in 4 min (NS-PA) and 2 min (NS-PB, NS-PC) respectively with a constant velocity. An ultrasonic probe was used; an interval of 15 s between each 15 s ultrasonic was. The power of the ultrasonic was 200 W. The speed of stirring was 800 rpm/min. 

To remove THF, freeze drying was conducted. Sucrose (3%, *w*/*v*) was added to nanosuspensions as protective agents. The nanosuspensions were frozen at −80 °C overnight and then put in the −30 °C freeze dryer (Christ α 2–4, Martin Christ, Osterode, Germany). The freeze drying process was −30 °C for 20 h, 0 °C for 4 h, and 20 °C for 4 h. The pressure was 0.2 mbar. Before use, the nanosuspensions were redispersed with 0.8% NaCl solution (10mM citrate-phosphate buffer, pH 7.0). 

NS-PA-L, NS-PB-L, and NS-PC-L were achieved by freeze drying from NS-PA, NS-PB, and NS-PC respectively. NS-PB-S and NS-PC-S were achieved by concentrating NS-PB and NS-PC to 30 mg/mL, first, and then freeze drying as the same process.

##### High-Pressure Homogenization Method

Tween-80 was dissolved into the bidistilled water. ZL-004 (30 mg/mL) was dispersed into water. Then the mixture was treated with the high-pressure homogenizer (Nano Debee, BEE International, South Easton, MA, USA). Particle size was measured at different time intervals during the process. NS-Hα (25,000 psi, 27 min, 0.5% Tween-80), NS-Hβ (25,000 psi, 12 min, 1.0% Tween-80), and NS-Hγ (15,000 psi, 27 min, 0.5% Tween-80) were prepared respectively.

NS-Hα-1 was achieved with 10 mM citrate-phosphate buffer (pH 7.0) and 0.8% NaCl added into NS-Hα. NS-Hα-2 was achieved with 0.15% PEG 4000, 10 mM citrate-phosphate buffer (pH 7.0) and 0.8% NaCl added into NS-Hα. 

#### 3.2.2. Characterization

##### DSC

A differential scanning calorimeter (Q2000, TA, New Castle, DE, USA) was used to measure the melting point. Samples were heated from 40 °C to 260 °C, at a rate of 10 K/min. 

##### XRPD

Nanosuspensions were dried and analyzed by an X-ray diffractometer (Bruker AXS, D8 Advance, Karlsruhe, Germany) with Cu (40 kV, 40 mA, λ = 1.54056 Å).

##### SEM

Nanosuspensions were dried at room temperature and coated with Au. A scanning electron microscope (S-3400N, Hitachi, Tokyo, Japan) was used to observe the shape and the size of the nanosuspension.

##### Particle Size and Zeta Potential

A laser diffraction particle sizing instrument (LA920, Horiba, Kyoto, Japan) was used to measure the particle size and distribution of nanosuspensions. D_50_ is the median size that splits the distribution with 50% below the diameter for volume distribution. Span was used to describe the distribution width. Transmittance was 85%–90%. Method validations met the requirements. The zeta potential of nanosuspensions was measured by Zetasizer Nano ZS 90 (Malvern, Worcestershire, UK)

#### 3.2.3. In Vitro Release Study

A drug release test was conducted on the dissolution tester (708 DS, 850 DS, Agilent, Singapore) according to apparatus П, API (D_50_ 20 μm), NS-PA-L, NS-PB-L, and NS-PC-L containing 6 mg ZL-004 were dispersed into the medium. The release medium was 900 mL (pH 7.4, 50 mM PBS, 0.1% SDS). The temperature was maintained at 37 °C. The speed of the paddle was 50 rpm/min. The drug content was analyzed at 231 nm with a UV detector by HPLC (Shimadzu, Kyoto, Japan). The sample was analyzed by a C18 column (5 μm, 4.6 × 250 mm, XTerra, Waters, Milford, MA, USA). The mobile phase was formed by 40% water (10 mM phosphate, pH 2.5) and 60% acetonitrile (*v*/*v*). The flow rate was 1.0 mL/min.

#### 3.2.4. In Vivo Pharmacokinetic Study

Thirty-two male SD rats were divided into 8 groups randomly. The oral administration group was administrated to intragastrically by suspending API in water with 0.5% CMC-Na and 4% Tween-80. Nanosuspensions were injected intramuscularly into the hind leg, behind the femur. NS-PA-L, NS-PB-L, and NS-PC-L were administrated 0.5 mL, NS-PB-S, NS-PC-S, NS-Hα-1, and NS-Hα-2 were administrated 0.1 mL. All of the dose levels were 10 mg/kg for ZL-004. Retro-orbital blood samples (0.3 mL) were collected after administration at different intervals and then centrifuged (12,000 rpm, 5 min) immediately to obtain plasma (0.1 mL). The plasma samples were stored at −20 °C until analysis.

Three-fold methanol was added to the plasma sample to sediment protein. The supernatant was diluted 10 fold with the mobile phase for analysis. ZL-004 was analyzed by Shim-pack XR-ODS (2.2 μm, 3.0 × 75 mm, Shimadzu, Kyoto, Japan) column, which was determined by HPLC-MS (LCMS-8030, Shimadzu, Kyoto, Japan). The mobile phase consisted of 20% water (*v*/*v*) with 10 mM ammonium formate and 0.01% formic acid (*v*/*v*), and 80% methanol (*v*/*v*); the flow rate was 0.3 mL/min. The detection was conducted in positive ionization mode with an electrospray ionization interface. Multiple-reaction monitoring was optimized for *m*/*z* 381 → 309 (ZL-004) and 256 → 157 (diphenhydramine, internal standard). Desolvation gas was 15 L/min; nebulizer gas was 3 L/min; capillary temperature was 400 °C; capillary voltage was 3.0 kV; desolvation temperature was 250 °C. method validations met the requirements. 

Pharmacokinetic parameters were calculated with DAS software (2.1.1, BioGuider Co., Shanghai, China) with a non-compartmental approach, and the maximum plasma of drug concentration (C_max_), time to maximum concentration (T_max_), area under plasma concentration-time curve (AUC), the terminal elimination half-life (t_1/2_) were achieved.

#### 3.2.5. Physical Stability

Nanosuspensions were stored at room temperature for 3 months. The particle size was analyzed after 1 month and 3 months of storage. Redispersity was investigated by hand-shaking for a minimum of 10 s.

#### 3.2.6. The Content of Dissolved Drug

The nanosuspensions were centrifuged (12,000 rpm, 20 min) to obtain the supernatant, and then the supernatant was filtered with the filter membrane with 0.1 um pore diameter. The sample was analyzed by a C18 column (5 μm, 4.6 × 250 mm, XTerra, Waters, Milford, MA, USA) at 231 nm with a UV detector by HPLC system (Shimadzu, Kyoto, Japan). The mobile phase was formed by 40% water (10 mM phosphate, pH 2.5) and 60% acetonitrile (*v*/*v*). The flow rate was 1.0 mL/min.

#### 3.2.7. Significance Test

Unpaired t-test was utilized to compare variables between two groups. *p* < 0.05 was considered statistically significant, 0.05 < *p* < 0.1 was considered nominally or marginally significant. Statistical analyses were performed by GraphPad Prism version 6.04 (GraphPad Software, La Jolla, CA, USA).

## 4. Conclusions

The initial release played an important part in the in vivo behavior of extended-release nanosuspensions, it was influenced by the content of dissolved drug significantly. Expect for the particle size, the content of dissolved drug was affected by the amount of free stabilizers. Therefore, NS-PC-L with larger particle size achieved higher C_max_ and similar AUC unexpectedly compared with NS-PB-L due to the higher free Tween-80 concentration. In addition, in order to achieve lower initial release for avoiding potential side effects, the content of dissolved drug was reduced by smaller administration volume of NS-PB-S and NS-PC-S. Meanwhile, NS-PC-S achieved more therapeutically effective days owing to the larger particle size. Furthermore, the crystal forms and in vivo behaviors of ZL-004 nanosuspensions prepared by the precipitation method (NS-PC-S) and high-pressure homogenization (NS-Hα-1) were similar. Additional 0.15% PEG 4000 (NS-Hα-2) enhanced the redispersity and stability of particle size for storage of 3 months at room temperature. Finally, an extended-release intramuscular ZL-004 nanosuspension for large-scale production with desired initial release and steady plasma concentration for about 1 month was achieved, which had good physical and chemical stability for 3 months. Bioavailability and t_1/2_ enhanced 161.2 fold and 22.9 fold compared with oral administration.

## Figures and Tables

**Table 1:** The particle size of NS-PA, NS-PB, NS-PC, and NS-Hα (Mean ± SD, *n* = 3).

Formulation	D_10_ (nm)	D_50_ (nm)	D_90_ (nm)
NS-PA	284 ± 27	424 ± 55	598 ± 98
NS-PB	459 ± 13	812 ± 48	1647 ± 166
NS-PC	645 ± 6	1185 ± 36	2249 ± 201
NS-Hα	591 ± 17	1134 ± 33	2518 ± 150

**Table 2:** Preparation, concentrations, and particle sizes.

Formulation	Preparation ^a^	Concentration (mg/mL)	D_10_ (nm)	D_50_ (nm)	D_90_ (nm)
**NS-PA-L**	freeze drying	6	274	397	558
**NS-PB-L**	freeze drying	6	472	836	1395
**NS-PC-L**	freeze drying	6	598	1156	2262
**NS-PB-S**	concentrating, freeze drying	30	448	806	1612
**NS-PC-S**	concentrating, freeze drying	30	618	1225	2466
**NS-Hα-1**	none	30	624	1211	2701
**NS-Hα-2**	adding 0.15% PEG 4000	30	627	1144	2402

^a^ adjusting pH and osmotic pressure were conducted for each formulation.

**Table 3:** Pharmacokinetic parameters of oral administration (Mean ± SD, *n* = 4).

Formulation	T_max (0–72 h)_ (h)	C_max (0–72 h)_ (ng/mL)	AUC_0→t_ (µg·h/mL)	t_1/2_ (h)
**Oral**	4.9 ± 0.8	48.6 ± 12.1 ^d^	0.6 ± 0.2 ^d^	11.0 ± 1.9 ^d^
**NS-PA-L**	14.5 ± 0.0	1104.7 ± 162.5 ^a^	192.3 ± 21.8 ^a^	170.5 ± 16.7 ^a^
**NS-PB-L**	14.5 ± 0.0	518.9 ± 109.7 ^a,†,b^	153.2 ± 20.2 ^a,b^	222.0 ± 23.3 ^a,b^
**NS-PC-L**	14.5 ± 0.0	685.5 ± 60.9 ^†,c^	163.0 ± 29.7 ^c^	406.8 ± 34.2 ^b^
**NS-PB-S**	7.0 ± 2.0	189.4 ± 32.5 ^b^	98.5 ± 21.2 ^b^	208.8 ± 17.4 ^†^
**NS-PC-S**	9.0 ± 2.0	232.9 ± 122.2 ^c^	110.2 ± 32.1 ^c^	266.9 ± 44.4 ^†^
**NS-Hα-1**	6.0 ± 0.0	218.5 ± 31.9	113.1 ± 14.5	241.5 ± 39.2
**NS-Hα-2**	7.0 ± 2.0	209.7 ± 24.2 ^d^	96.9 ± 9.8 ^d^	252.3 ± 48.8 ^d^

^†^*p* < 0.1, ^a,b,c,d^ *p* < 0.05.

## Supplementary Materials

The following are available online. Figure S1: Effect of homogenization time, pressure, and concentration of Tween-80 on the particle size, Figure S2: DSC curves of NS-PA, NS-PB, NS-PC, NS-Hα, and API (a). XPRD curves of NS-PC, NS-Hα, and API (b), Table S1: The particle sizes of NS-Hα-1 and NS-Hα-2 during storage of 3 months at room temperature (Mean ± SD, *n* = 3).
