# Supplementary material for: Optimization of Extended-Release ZL-004 Nanosuspensions for In Vivo Pharmacokinetic Study to Enhance Low Solubility and Compliance
Source: Molecules. 2018 Dec 20;24(1):7. doi: 10.3390/molecules24010007 (PMC6337511; doi:10.3390/molecules24010007)
Supplement: Supplementary file 1 [file molecules-24-00007-s001.pdf]

Table S1. The particle sizes of NS-H-1α and NS-Hα-2 during storage of 3 months at room temperature (Mean ± SD, *n*=3).

Formulation	Time (month)	Redispersity	D_{10} (nm)	D_{50} (nm)	D_{90} (nm)
NS-Hα-2	0	Yes	586± 17	1213± 81	2303± 58
	1	Yes	595± 34	1178± 67	2496± 42
	3	Yes	623±35	1237±90	2640±63
NS-Hα-1	0	Yes	569±8	1150± 17	2761± 149
	1	Yes	621± 47	1292±42	2663± 138
	3	No	893±95	1506+174	3149±226

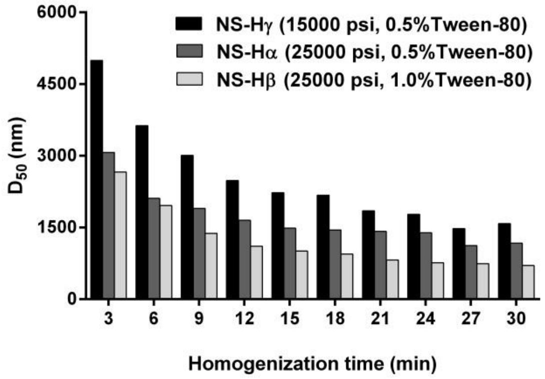

Fig.S1. Effect of homogenization time, pressure, and concentration of Tween-80 on the particle size.

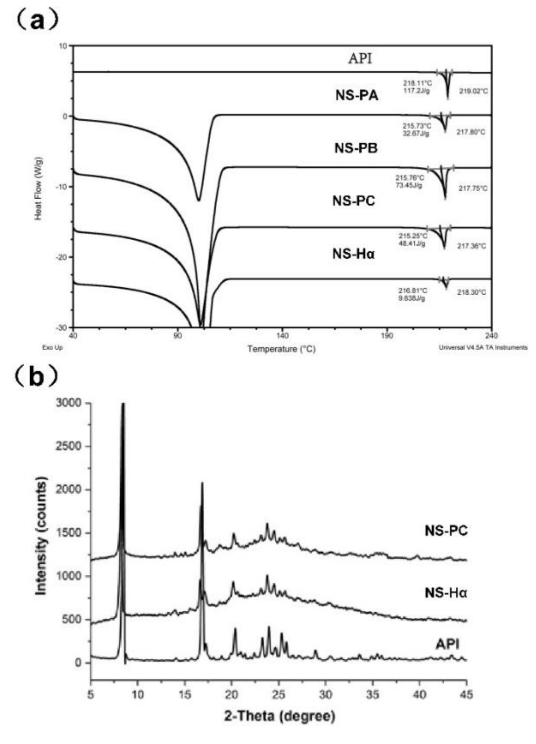

Figure S2. DSC curves of NS-PA, NS-PB, NS-PC, NS-Hα, and API (a). XPRD curves of NS-PC, NS-Hα, and API (b).
